# Comparative Efficacy and Safety of Conventional Dresden, Transepithelial, and Accelerated Corneal Collagen Cross-Linking Protocols for Progressive Keratoconus: A Systematic Review

**DOI:** 10.7759/cureus.102911

**Published:** 2026-02-03

**Authors:** Bradley A Nordin

**Affiliations:** 1 Ophthalmology, Huffman and Huffman Eye Physicians and Surgeons, London, USA

**Keywords:** accelerated cxl, corneal collagen cross-linking, dresden protocol, endothelial safety, epi-on/off cxl, keratoconus, kmax, systematic review, transepithelial cxl, visual acuity

## Abstract

Objective: The objective of this study was to compare the efficacy and safety of conventional Dresden, transepithelial (epithelium-on), and accelerated corneal collagen cross-linking (CXL) protocols for the treatment of progressive keratoconus.

Methods: A systematic review of 84 studies was conducted in accordance with Preferred Reporting Items for Systematic reviews and Meta-Analyses (PRISMA) 2020 guidelines. Study screening and data extraction were supported by AI-assisted tools with full manual verification. Extracted outcomes included keratometric stabilization, visual acuity, endothelial cell density (ECD), complications, corneal thickness changes, and biomechanical or surrogate markers.

Results: Conventional epithelium-off (epi-off) CXL demonstrated the most consistent long-term keratometric stabilization and visual acuity preservation, with mean Kmax flattening of approximately 1.0-2.3 D at 12-36 months and durability extending up to five years in long-term datasets, alongside corrected distance visual acuity improvements of approximately 0.10-0.23 logMAR. Accelerated CXL protocols achieved comparable short-term outcomes (approximately 0.8-1.5 D Kmax flattening at 6-12 months) but exhibited greater variability in durability at longer follow-up. Standard transepithelial approaches generally produced smaller effects, although enhanced epi-on protocols incorporating oxygen supplementation or modified riboflavin delivery achieved keratometric stabilization approaching epi-off outcomes in selected studies (approximately 1.5-1.7 D flattening). Across all protocols, endothelial cell density was preserved, with changes typically within physiologic variability (<5%). Transient corneal haze occurred more frequently following conventional epi-off CXL (approximately 40-70%) than accelerated protocols (approximately 20-47%), while serious complications, including infectious keratitis, remained rare (approximately 0.001-0.5%).

Conclusion: Conventional epi-off CXL has the strongest evidence for durable keratometric stabilization and visual acuity preservation in progressive keratoconus. Accelerated protocols offer similar short-term efficacy with improved treatment efficiency, while enhanced transepithelial approaches may be appropriate for selected patients prioritizing reduced invasiveness and postoperative discomfort, with potential trade-offs in long-term durability.

## Introduction and background

Background on keratoconus

Keratoconus is a progressive, non-inflammatory ectatic disorder of the cornea characterized by thinning and conical protrusion, leading to irregular astigmatism, myopia, and significant visual impairment. Epidemiologically, the condition typically manifests in adolescence or early adulthood, with a pooled global prevalence of 289.1 cases per 100,000 individuals (0.29%) and an incidence of 4.0 per 100,000 person-years, affecting over 23.7 million people worldwide and showing increasing trends over time, particularly post-2020. The highest prevalence and incidence are observed in the 20-29 age group, with rates of 525.5 per 100,000 and 20.8 per 100,000 person-years, respectively, highlighting a substantial burden in young adults aged 18-39 years across diverse regions [1].

In terms of progression, keratoconus advances with increasing corneal steepening, as evidenced by an average increase in maximum keratometry (Kmax) of 0.7 diopters (D) over 12 months, with faster rates in younger patients and those with baseline Kmax steeper than 55 D. This natural history leads to deteriorating visual acuity and quality of life, often culminating in severe disability if unchecked [2]. The need for stabilization is critical to halt this progression, prevent the requirement for advanced interventions like corneal transplantation, and preserve vision, particularly in high-risk groups where closer monitoring and early treatments like corneal cross-linking are recommended [1,2].

Overview of CXL

Corneal collagen cross-linking (CXL) represents a paradigm shift in keratoconus management, as it is the only minimally invasive treatment proven to halt disease progression by biomechanically strengthening the corneal stroma [3]. The mechanism involves the application of riboflavin (vitamin B2) as a photosensitizer, followed by ultraviolet-A (UV-A) irradiation at a wavelength of 365-370 nm to generate reactive oxygen species, which induce covalent bonds between collagen fibrils and proteoglycans through oxidative (Type 1 and Type 2) and glycosylation pathways, thereby increasing corneal rigidity and resistance to ectatic changes [3,4]. Oxygen plays a crucial role in this photochemical reaction, and riboflavin also acts as an optical buffer by absorbing UV-A photons and converting them into harmless chemical energy (via photochemical excitation and subsequent reactive oxygen species generation), thereby protecting posterior ocular structures like the endothelium, lens, and retina from UV damage during the procedure [3,5].

The procedure evolved from foundational studies in the late 1990s at the University of Dresden, where initial experiments demonstrated increased corneal rigidity through riboflavin/UV-A-induced cross-links [5,6]. The conventional "Dresden protocol," an epithelium-off (epi-off) approach introduced in 2003, involves epithelial debridement of the central 7-9 mm cornea, riboflavin (0.1% in 20% dextran) instillation every two to five minutes for 30 minutes, and UV-A exposure at 3 mW/cm² for 30 minutes, delivering a total energy dose of 5.4 J/cm² [3,4]. Transepithelial (epi-on) protocols preserve the epithelium to minimize complications, enhancing riboflavin penetration via chemical enhancers (e.g., benzalkonium chloride), iontophoresis, or supplemental oxygen, offering reduced discomfort but potentially lower long-term efficacy compared to epi-off methods unless modified [3,7]. To address limitations such as prolonged treatment time, postoperative pain, and infection risk, accelerated variants emerged, increasing UV-A irradiance (e.g., 9-30 mW/cm²) while shortening duration (e.g., 9 mW/cm² for 10 minutes) based on Bunsen-Roscoe's law of reciprocity (which posits that the photochemical effect is proportional to light intensity × exposure time, applicable at moderate but not extreme intensities), though efficacy may vary due to reduced oxygen availability at higher intensities [3,5].

Rationale and scope

Despite numerous systematic reviews on CXL, significant gaps persist, including limited head-to-head comparisons of protocols, inconsistent long-term data beyond five years, and heterogeneous definitions of progression and stabilization [8,9]. Prior syntheses often focus on short-term efficacy in isolation, with fewer addressing comprehensive safety profiles or biomechanical surrogates in diverse patient groups, such as non-pediatric populations, where most studies emphasize pediatric cases due to aggressive progression [8,10]. With emerging 2025 data from registries and trials, an updated review is warranted to guide clinical decision-making amid evolving protocols [9].

The objective of this systematic review is to address the following PICO question: In patients with progressive keratoconus (P), what is the comparative efficacy and safety (O) of accelerated CXL, conventional (Dresden protocol) CXL, and transepithelial (epi-on) CXL (I) versus each other (C) with respect to stabilization of keratometric indices, improvement in uncorrected and corrected distance visual acuity, preservation of endothelial cell density, and rates of serious complications (haze, persistent edema, infection, scarring) at ≥12 months follow-up?

## Review

Materials and methods

Study Design

This systematic review was conducted in accordance with the Preferred Reporting Items for Systematic Reviews and Meta-Analyses (PRISMA) 2020 guidelines [11]. A qualitative synthesis was performed, including randomized controlled trials (RCTs), prospective and retrospective cohort studies, observational registries, and meta-analyses evaluating corneal CXL protocols in progressive keratoconus. Quantitative meta-analysis was not undertaken due to heterogeneity in study design, protocol parameters, outcome definitions, and follow-up durations.

The review protocol was not prospectively registered. This decision reflected the exploratory and comparative nature of evolving corneal cross-linking protocols and the absence of standardized long-term outcome frameworks across study designs.

Search Strategy

A comprehensive literature search was conducted between December 21 and December 26, 2025, using PubMed/MEDLINE (Medical Literature Analysis and Retrieval System Online), Embase, Cochrane Central Register of Controlled Trials (CENTRAL), Scopus, and ClinicalTrials.gov. Searches covered studies published from January 1, 2013, through December 26, 2025. No initial language restrictions were applied; non-English articles were translated when relevant. Grey literature and reference lists of recent systematic reviews and meta-analyses (e.g., Deshmukh et al., 2023 [9]; Kobashi et al., 2020 [12]) were manually screened to identify additional eligible studies.

Detailed database-specific search strategies and yields are presented in Table 1.

**Table 1:** Detailed search strategies and yields

Database/Source	Search Query	Filters/Limits	Date Searched	Number of Results
PubMed/MEDLINE	(keratoconus[MeSH Terms] OR keratoconus[Title/Abstract] OR "keratoconus"[Title/Abstract]) AND ("Cross-Linking Reagents/therapeutic use"[MeSH Terms] OR "corneal collagen cross-linking"[Title/Abstract] OR "corneal cross-linking"[Title/Abstract] OR CXL[Title/Abstract] OR "cross linking"[Title/Abstract] OR "collagen cross linking"[Title/Abstract] OR "riboflavin UVA"[Title/Abstract] OR "riboflavin ultraviolet"[Title/Abstract] OR "photoactivated chromophore"[Title/Abstract]) AND (accelerated[Title/Abstract] OR conventional[Title/Abstract] OR Dresden[Title/Abstract] OR "transepithelial"[Title/Abstract] OR "epi-on"[Title/Abstract] OR "epi off"[Title/Abstract] OR "epithelium-off"[Title/Abstract] OR "epithelium-on"[Title/Abstract] OR pulsed[Title/Abstract] OR customized[Title/Abstract] OR iontophoresis[Title/Abstract] OR protocol*[Title/Abstract])	Humans; Article types: Clinical Trial, Randomized Controlled Trial, Comparative Study, Observational Study; Publication dates: 2013 onward	January 1, 2013 to December 26, 2025	55
Embase	#1: 'keratoconus'/exp OR keratoconus:ti,ab,kw #2: ('corneal collagen cross linking'/exp OR 'cross linking'/exp OR cxl:ti,ab,kw OR "corneal cross-linking":ti,ab,kw OR "collagen cross linking":ti,ab,kw OR "riboflavin UVA":ti,ab,kw OR "riboflavin ultraviolet":ti,ab,kw OR "photoactivated chromophore":ti,ab,kw) #3: (accelerated:ti,ab,kw OR conventional:ti,ab,kw OR dresden:ti,ab,kw OR transepithelial:ti,ab,kw OR "epi-on":ti,ab,kw OR "epi off":ti,ab,kw OR "epithelium-off":ti,ab,kw OR "epithelium-on":ti,ab,kw OR pulsed:ti,ab,kw OR customized:ti,ab,kw OR iontophoresis:ti,ab,kw OR protocol*:ti,ab,kw) #4: #1 AND #2 AND #3	Humans; Evidence-based medicine (e.g., RCTs, controlled trials); Publication types: articles, conference abstracts; Dates: 2013 to December 2025	January 1, 2013 to December 26, 2025	65
Cochrane CENTRAL	Adapted queries emphasizing comparative terms and protocol variants (similar to PubMed and Embase)	Humans; Clinical trials and comparative studies; Publication dates: 2013 onward	January 1, 2013 to December 26, 2025	28
Scopus	Adapted queries emphasizing comparative terms and protocol variants (similar to PubMed and Embase)	Humans; Articles, reviews, conference papers; Publication dates: 2013 onward	January 1, 2013 to December 26, 2025	43
Grey Literature (ClinicalTrials.gov)	Keywords: "keratoconus cross-linking comparison"	No specific filters; Focused on ongoing and completed trials	January 1, 2013 to December 26, 2025	4
Hand-Searching	Reviewing reference lists of recent reviews and meta-analyses (e.g., Deshmukh et al., 2023 [9]; Kobashi et al., 2020 [12])	N/A	N/A	5
Deduplication	N/A	N/A	N/A	34 duplicates removed
Overall (Post-Deduplication)	Combined from all databases, exported in NBIB format	N/A	N/A	166 unique sources

Study Selection

Study selection was conducted in two stages: title and abstract screening followed by full-text eligibility assessment. Predefined inclusion and exclusion criteria, including population characteristics, study design, intervention protocols, comparators, outcome measures, and minimum follow-up duration, are detailed in Appendix A.

AI-assisted tools were used as an adjunct during the title and abstract screening phase to support organization and prioritization of records potentially meeting eligibility criteria. AI outputs were used solely to enhance screening efficiency and did not independently determine study eligibility. All records identified as potentially relevant underwent direct manual review.

Full-text articles were assessed by the author for eligibility using the predefined criteria, and all final inclusion and exclusion decisions were made following manual evaluation of the full texts.

Data Extraction

Data extraction was performed using standardized extraction templates capturing study design, population characteristics, cross-linking protocols, outcome measures, and follow-up duration. Extracted outcomes included keratometric parameters, visual acuity measures, endothelial cell density (ECD), corneal thickness changes, biomechanical or surrogate markers, and reported complications.

AI-assisted tools were used to support structured data extraction based on predefined data fields and extraction instructions, as detailed in Appendix B. All extracted data were manually verified against the original publications prior to synthesis. To enhance reliability, approximately 20% of included studies underwent secondary verification, with extracted values cross-checked against source texts and discrepancies resolved by direct reference to the original articles. No AI-assisted output was incorporated into the analysis without author verification.

Risk of Bias Assessment

Risk of bias was assessed manually using the Cochrane Risk of Bias 2 (RoB 2) tool for RCTs and the Risk Of Bias In Non-randomized Studies - of Interventions (ROBINS-I) tool for non-randomized studies [13]. Assessments were based on reported study methodology, outcome measurement, and completeness of follow-up. AI tools were not used to assign or automate risk-of-bias judgments.

Data Synthesis

Extracted data were synthesized qualitatively and grouped by outcome domain, including keratometric stabilization, visual acuity outcomes, endothelial safety, corneal thickness changes, biomechanical or surrogate measures, and complications. Results were stratified by cross-linking protocol to facilitate comparative interpretation. Representative ranges and medians were reported descriptively where appropriate. Formal meta-analysis was not performed due to methodological heterogeneity across included studies.

Statistical Reporting Conventions

Where reported, p-values and 95% confidence intervals (CIs) reflect statistical testing performed in the original source studies and are presented as reported by individual randomized controlled trials, observational cohorts, registries, or meta-analyses. No additional pooled statistical analyses were performed for this review. Continuous outcomes are reported as mean ± standard deviation (SD) when available, or as median (interquartile range, IQR) where distributions were nonparametric or reported as such in the source studies. Units are reported uniformly across tables and text.

Results

**Figure 1:**
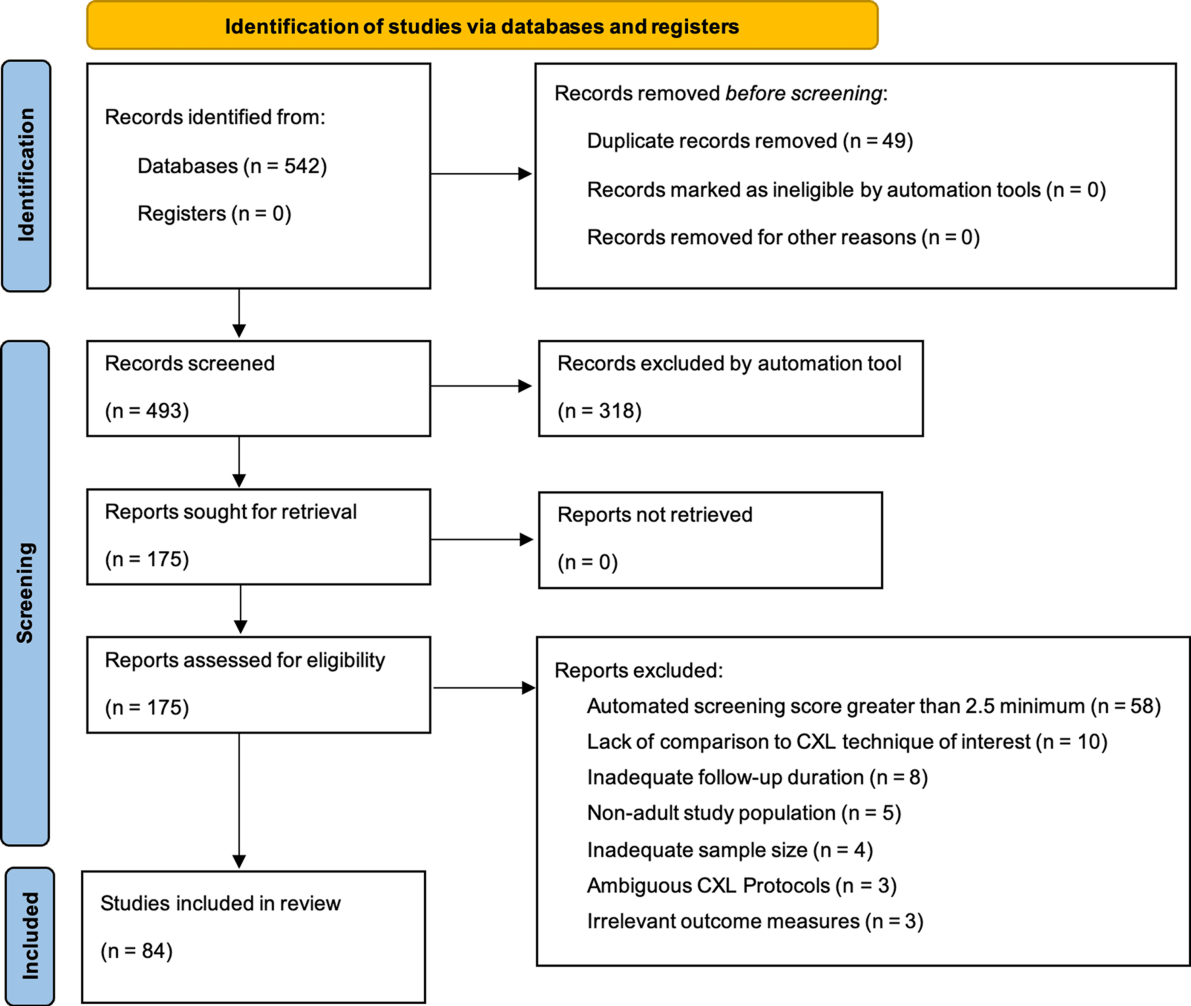
PRISMA flowchart showing study selection process CXL: corneal collagen cross-linking; LLM: large language model; PRISMA: Preferred Reporting Items for Systematic Review and Meta-Analysis

Following database searching, 542 records were identified (166 from manual searches and 376 from automated database queries). After the removal of 49 duplicate records, 493 unique records proceeded to title and abstract screening. As a result of title and abstract screening, 318 records were excluded, and 175 reports were retrieved for full-text assessment. Of these, 91 full-text articles were excluded. Ultimately, 84 studies met the inclusion criteria and were included in the qualitative synthesis. The study selection process is summarized in the PRISMA flow diagram (Figure 1).

Characteristics of Included Studies

The 84 included studies comprised a heterogeneous mix of randomized controlled trials, non-randomized comparative studies, observational cohorts, registry-based analyses, systematic reviews, and non-clinical investigations. Conventional epi-off (Dresden) protocols were the most extensively studied and included multiple prospective randomized controlled trials, large observational cohorts, and long-term registry studies with follow-up extending up to ten years. Transepithelial (epi-on) protocols were evaluated primarily in prospective randomized and non-randomized comparative studies, supplemented by several observational cohorts and one experimental laboratory (ex vivo) investigation assessing riboflavin stromal penetration. Accelerated cross-linking protocols were examined across a broad spectrum of designs, including randomized controlled trials, non-randomized comparative studies, retrospective cohorts, and registries, with follow-up ranging from short-term (six to 12 months) to intermediate and long-term intervals of up to five years. In addition, two systematic reviews and meta-analyses and one economic modeling study were included to contextualize comparative efficacy and cost-effectiveness but were not treated as primary clinical evidence. Key study characteristics, including design, protocol type, sample size, and follow-up duration, are summarized in Table 2.

**Table 2:** Summary characteristics of included studies (N=84) A-CACXL: accelerated contact lens-assisted corneal cross-linking; A-CXL: accelerated corneal cross-linking; ATE-CXL: accelerated transepithelial corneal cross-linking (a high-intensity, short-duration epi-on protocol); cl-ACXL: continuous light-accelerated corneal cross-linking; CLCXL:  contact lens-assisted corneal cross-linking (a modified accelerated protocol using a contact lens to aid in treatment for thin corneas); cCXL: customized corneal cross-linking; CRXL: corneal cross-linking with mechanical compression (an accelerated protocol involving mechanical compression of the cornea using a flat rigid contact lens during CXL); CXL: corneal collagen cross-linking; EFPL-M-TECXL: transepithelial enhanced fluence pulsed light accelerated CXL; Epi-off: epithelium-off protocols (conventional Dresden method); Epi-on: epithelium-on or transepithelial protocols, preserves the corneal epithelium; Epioxa™: FDA-approved epi-on CXL using oxygen-enriched RF for keratoconus; EpiSmart: investigational epi-on CXL with RiboStat (RF + sodium iodide); eRF: enhanced riboflavin; I-CXL: transepithelial corneal collagen crosslinking using iontophoresis (riboflavin delivery via iontophoresis); NR: not reported; NRCT: non-randomized comparative trial; pl-ACXL: pulsed light-accelerated corneal cross-linking; PRK: photorefractive keratectomy; RCT: randomized controlled trial; RF: riboflavin (the photosensitizer used in CXL protocols); STARE-X: selective transepithelial topography-guided photorefractive keratectomy combined with simultaneous accelerated corneal crosslinking; T-CAT: topography-guided customized ablation treatment (a refractive procedure combined with CXL); TA-CXL: topo-pachimetric accelerated epi-on CXL (customized based on topography and pachymetry); TE-CXL: transepithelial corneal cross-linking (an epi-on variant preserving the epithelium)

Protocol Group	Study	Study Design	CXL Protocol(s)	Sample Size	Follow-up Duration
Conventional Dresden / Standard Epi-off	Alqudah et al., 2025 [14]	Retrospective observational cohort	Dresden vs I-CXL	107 patients	1 year
	Bhattacharyya et al., 2019 [15]	Prospective observational cohort	Dresden vs Control	78 eyes	6 months
	Choi et al., 2017 [16]	Prospective RCT	Dresden vs A-CXL	28 eyes	6 months
	Danesh et al., 2021 [17]	Prospective observational cohort	Dresden	31 eyes	1 year
	Dervenis et al., 2020 [18]	Retrospective case series	Dresden vs A-CXL	59 eyes	7 months
	Elghobaier et al., 2025 [19]	Retrospective case series	Dresden	223 eyes	3 years
	Elmassry et al., 2021 [20]	Retrospective observational cohort	Dresden vs TE-CXL vs A-CXL	6120 eyes	10 years
	Ferdi et al., 2019 [21]	Systematic review and meta-analysis	Epi-off (various)	41 studies	1 year
	Ferdi et al. (Registry), 2023 [2]	Observational registry study	Dresden	162 eyes	5 years
	Gustafsson et al., 2025 [22]	Prospective RCT (non-inferiority)	Iso vs Hypo-osmolar RF	54 patients	1 year
	Hallahan et al., 2014 [23]	Prospective RCT	Dresden	51 eyes	3 months
	Kandel et al. (Registry), 2024 [24]	Observational registry study	Dresden	176 eyes	5 years
	Kymionis et al., 2016 [25]	Prospective NRCT	Dresden vs cCXL	32 eyes	1 month
	Lindstrom et al., 2021 [26]	Economic modeling study	Dresden vs no CXL	Simulated cohort	Lifetime
	Marafon et al., 2020 [27]	Retrospective comparative cohort	Dresden vs A-CXL	113 eyes	Mean 37.6 months
	Padmanabhan et al., 2014 [28]	Prospective NRCT	Dresden vs T-CAT+CXL	27 eyes	Mean 7.7 months
	Price et al., 2018 [29]	Prospective interventional case series	Dresden vs A-CXL	644 eyes	Median 3.5 years
	Rosenblat et al., 2014 [30]	Prospective NRCT	Standard vs hypotonic RF	39 patients	1 year
	Rosenblat et al., 2016 [31]	Prospective RCT	Standard vs hypotonic RF	48 eyes	1 year
	Seyedian et al., 2015 [32]	Prospective RCT	Dresden vs Control	52 eyes	1 year
	Soeters et al., 2014 [33]	Retrospective observational cohort	Dresden	119 eyes	1 year
	Soeters et al., 2015 [7]	Prospective RCT	Dresden vs TE-CXL	39 eyes	1 year
	Strmeňová et al., 2015 [34]	Retrospective observational cohort	Dresden	88 eyes	2 years
	Vandevenne et al., 2023 [35]	Prospective RCT (non-inferiority)	Dresden vs cCXL	124 patients	1 year
	Wittig-Silva et al., 2014 [36]	Prospective RCT	Dresden vs Control	100 eyes	3 years
Transepithelial / Epi-on	Al Fayez et al., 2015 [37]	Prospective RCT	TE-CXL vs Dresden	70 eyes	Mean 40 months
	APRICITY-A, 2023 [38]	Prospective RCT	EpiSmart vs Sham	400 subjects	1 year
	APRICITY-B, 2023 [39]	Prospective RCT	EpiSmart vs Sham	400 subjects	1 year
	Bikbova et al., 2016 [40]	Prospective RCT	I-CXL vs Dresden	149 eyes	2 years
	Caruso et al., 2016 [41]	Prospective NRCT	TE-CXL	25 eyes	2 years
	Caruso et al., 2021 [42]	Prospective RCT	TA-CXL vs Dresden	NR	2 years
	Cassagne et al., 2014 [43]	Prospective interventional case series	I-CXL	NR	NR
	Cifariello et al., 2018 [44]	Prospective RCT	TE-CXL vs Dresden	40 eyes	2 years
	Epstein et al., 2022 [45]	Prospective RCT	EpiSmart vs Control	1922 subjects	1 year
	Ferrini et al., 2023 [46]	Prospective observational cohort	TE-CXL vs Dresden	NR	NR
	Gatzioufas et al., 2016 [47]	Prospective observational cohort	TE-CXL	26 eyes	1 year
	Lesniak et al., 2014 [48]	Prospective RCT	TE-CXL	30 eyes	6 months
	Lombardo et al., 2016 [49]	Prospective RCT	I-CXL vs Dresden	34 eyes	6 months
	Lombardo et al., 2017 [50]	Prospective RCT	I-CXL vs Dresden	34 eyes	2 years
	Mastropasqua et al., 2014 [51]	Experimental laboratory (ex vivo) study	I-CXL vs TE-CXL vs Dresden	10 donor corneas	Immediate (procedural)
	Mazzotta et al., 2020 [52]	Prospective interventional case series	ATE-CXL + O2	27 eyes	6 months
	Mazzotta et al., 2022 [53]	Prospective NRCT	EFPL-M-TECXL + eRF	40 eyes	3 years
	Rechichi et al., 2021 [54]	Prospective NRCT	STARE-X	100 eyes	≥2 years
	Shao et al., 2020 [55]	Prospective observational cohort	TE-CXL	46 eyes	6 months
	Shetty et al., 2014 [56]	Retrospective case series	TE-CXL vs Dresden vs A-CXL (epi-off)	2350 patients	≥6 months
	Smith et al., 2025 [57]	Prospective RCT	Epioxa™ vs Sham	312 eyes	1 year
	Stojanovic et al., 2014 [58]	Prospective RCT	TE-CXL vs Epi-off	40 eyes	Median 2 years
	Stulting et al., 2018 [59]	Prospective observational cohort	TE-CXL	512 eyes	2 years
	Sun et al., 2018 [60]	Prospective NRCT	TE-CXL + O2	26 eyes	1 year
	Vinciguerra et al., 2016 [61]	Prospective NRCT	I-CXL vs Dresden	34 eyes	1 year
Accelerated	Abdel-Radi et al., 2023 [62]	Prospective interventional case series	A-CXL	45 eyes	6 months
	Adapted Fluence Study, 2015 [63]	Prospective RCT	A-CXL (7 vs 10 min)	40 patients	1 year
	Ang et al., 2025 [64]	Retrospective observational study	A-CXL	70 eyes	1 year
	Asgari et al., 2018 [65]	Prospective NRCT	A-CXL (18 vs 9 mW)	60 eyes	1 year
	Avni-Zauberman et al., 2021 [66]	Retrospective cohort	A-CXL vs Dresden	124 eyes	1 year
	Badawi, 2021 [67]	Retrospective cohort	A-CXL vs TE-CXL vs Dresden	104 eyes	1 year
	Bunin et al., 2025 [68]	Retrospective cohort	A-CXL vs A-CACXL	62 eyes	3 years
	Dina et al., 2025 [69]	NRCT (non-inferiority)	A-CXL vs Dresden	41 eyes	1 year
	Dongre et al., 2024 [70]	Retrospective case series	A-CXL vs Dresden	964 eyes	1 year
	Gupta et al., 2024 [71]	Retrospective comparative cohort	CACXL vs TE-CXL vs Dresden	94 eyes	1 year
	Hagem et al., 2017 [72]	Prospective RCT	A-CXL vs Dresden	40 eyes	1 year
	Hagem et al., 2019 [73]	Prospective RCT	A-CXL vs Dresden	40 eyes	2 years
	Hashemi et al., 2015 [74]	Prospective RCT	A-CXL vs Dresden	62 eyes	6 months
	Hashemi et al., 2017 [75]	Prospective interventional case series	A-CXL (18 vs 9 mW)	62 eyes	1 year
	Herber et al., 2018 [76]	Retrospective case series	A-CXL (beam profiles)	45 eyes	1 year
	Iqbal et al., 2019 [77]	Prospective NRCT	A-CXL+PRK vs Dresden	125 eyes	2 years
	Ishii et al., 2022 [78]	Prospective NRCT	ATE-CXL	34 eyes	3 years
	Kandel et al., 2021 [79]	Observational registry study	A-CXL vs Dresden	684 eyes	1 years
	Karotkar et al., 2022 [80]	Prospective RCT	pl-ACXL vs cl-ACXL	100 eyes	1 year
	Kobashi et al., 2020 [12]	Systematic review and meta-analysis	A-CXL vs Dresden	6 RCTs	1 year
	Kortuem et al., 2017 [81]	Retrospective comparative cohort	A-CXL vs Dresden	286 eyes	3 years
	Males et al., 2018 [82]	Retrospective comparative cohort	A-CXL vs Dresden	42 eyes	≥1 years
	Manumuraleekrishna et al., 2024 [83]	Prospective interventional case series	A-CXL (hypo- vs iso-osmolar RF)	100 eyes	1 year
	Mazzotta et al., 2021 [84]	Prospective NRCT	A-CXL	156 eyes	5 years
	Ng et al., 2016 [85]	Prospective NRCT	A-CXL vs Dresden	26 eyes	Mean 13.9 months
	Ozsaygili et al., 2021 [86]	Retrospective comparative cohort	A-CXL	64 eyes	1 year
	Recalde et al., 2019 [87]	Prospective observational cohort	A-CXL	22 eyes	1 year
	Rehnman et al., 2015 [88]	Prospective RCT	CRXL vs Dresden	60 eyes	6 months
	Sherif et al., 2016 [89]	Prospective interventional case series	pl-ACXL	20 eyes	Intraoperative
	Šklebar et al., 2025 [90]	Prospective observational cohort	A-CXL vs Dresden	38 eyes	9 months
	Tomita et al., 2014 [91]	Prospective NRCT	A-CXL vs Dresden	48 eyes	1 year
	Turhan et al., 2019 [92]	Prospective interventional case series	A-CXL	52 eyes	NR
	Turunç et al., 2025 [93]	Prospective interventional case series	A-CXL	113 eyes	6 months
	Yousif et al., 2023 [94]	Retrospective comparative cohort	pl-ACXL vs cl-ACXL	200 eyes	1 year

Risk of Bias

Among the 84 included studies, risk of bias varied by design, with randomized controlled trials (RCTs) generally demonstrating lower risk than observational studies. Of the 26 RCTs evaluated using RoB 2, 10 (38%) were judged to be at low overall risk [7,16,23,32,36,40,72-74,88], characterized by robust randomization procedures (e.g., computer-generated sequences with allocation concealment) and minimal deviations from intended interventions or missing data. The remaining 16 RCTs (62%) were assessed as having some concerns [22,31,35,37-39,42,44,45,48-50,57,58,63,80], most commonly due to unclear blinding of outcome assessors or moderate attrition (approximately 10-20% loss to follow-up without imputation). No RCTs were rated at high risk, although selective reporting concerns were identified in four trials with unregistered protocols [23,32,74,88].

Of the 54 non-randomized clinical studies assessed using ROBINS-I V2, eight (15%) were judged to be at low risk of bias [15,20,24,27,34,46,59,79], primarily comprising well-matched registries or cohorts with robust outcome ascertainment; 25 (46%) were at moderate risk [14,17-19,22,25,28-30,33,41,43,47,52-55,60,61,65-67,70,75,81], largely due to residual confounding (e.g., baseline differences in keratoconus severity) that was partially mitigated by study design or adjustment; 18 (33%) were at serious risk [56,62,64,68,69,71-73,76-78,82-86,90,91], most often related to selection bias or unaddressed missing data; and three (6%) were at critical risk [63,89,92], reflecting substantial intervention misclassification or outcome measurement limitations.

The two meta-analyses [12,21] were not formally appraised for risk of bias, instead inheriting the bias profile of their included randomized controlled trials, which were predominantly at low risk to some concerns. One experimental laboratory (ex vivo) study [51], which evaluated intrastromal riboflavin penetration across different imbibition techniques in donor corneas, was likewise not assessed using RoB 2 or ROBINS-I and is reported separately as mechanistic evidence. One economic modeling study [26], synthesizing clinical efficacy and cost assumptions from multiple clinical sources, was also excluded from formal bias assessment. Overall, while RCTs provided higher-quality evidence for short-term outcomes, non-randomized studies contributed important long-term data despite elevated risks of confounding and selection, influencing the strength of conclusions for accelerated and transepithelial cross-linking protocols.

Keratometric Outcomes

Overall, epi-off (conventional and accelerated) CXL protocols produced greater and more durable keratometric flattening than transepithelial approaches, with the most robust long-term stabilization observed after conventional Dresden CXL [2,15,16,32,34,36,58]. Accelerated protocols achieved comparable short-term Kmax reductions, while transepithelial outcomes were more variable unless enhanced with modified riboflavin delivery or supplemental oxygen [12,37,40,41,45,57].

Keratometric outcomes were primarily assessed through changes in maximum keratometry (Kmax), which were reported in 70 of the 84 included studies [2,7,12,14-42,44,45,47-50,52,57-61,63-67,69-75,77-81,83-85,87,88,91-94]. Across protocols, studies consistently demonstrated corneal flattening and stabilization, although the magnitude, onset, and durability of effect varied according to protocol type, baseline disease severity, and follow-up duration [7,12,21,24,27,36]. Long-term data beyond three years were available predominantly for conventional Dresden epi-off variants [2,20,24,27,36].

For conventional Dresden epi-off CXL, randomized trials, comparative cohorts, and large registry studies consistently demonstrated progressive Kmax flattening with durable stabilization across follow-up intervals, with reported p-values and confidence intervals derived from individual study-level analyses rather than pooled estimates [2,15,16,32,34,36,58]. Collectively, these findings support the superior long-term durability of conventional epi-off CXL for keratometric stabilization, while indicating that accelerated protocols offer comparable short-term efficacy with greater variability in long-term stability and that transepithelial approaches generally require protocol enhancements to achieve similar effects [12,24,37,40,45,57]. Quantitative Kmax changes are summarized in Tables 3-5 and are reported as mean ± SD or median [IQR], as specified by the source studies, with p-values reflecting within-study or between-group comparisons conducted by the original authors.

**Table 3:** Summary of keratometric outcomes (Kmax flattening, stabilization rate) from representative studies on conventional Dresden cross-linking protocols in progressive keratoconus A-CXL: accelerated corneal cross-linking; CXL: corneal collagen cross-linking; D: diopter; Kmax: maximum keratometry (the steepest corneal curvature measurement in diopters); NS: not significant (p-value > 0.05)

Study	CXL Protocol	Kmax Change	Comparison	Timepoint	Stabilization Rate
Wittig-Silva et al., 2014 [36]	Dresden	-1.03 ± 0.19 D (p < 0.001)	vs Control: +1.75 (p < 0.001)	3 years	100% vs 60%
Strmeňová et al., 2015 [34]	Dresden	-0.97 D (p < 0.01)	NR	2 years	97%
Seyedian et al., 2015 [32]	Dresden	-0.22 ± 0.6 D (p < 0.05)	vs Control: +0.41 D (p < 0.01)	1 year	88% vs 62%
Choi et al., 2017 [16]	Dresden	-0.55 ± 0.89 D (p < 0.05)	vs A-CXL: no change (p < 0.05)	6 months	100% vs 100%
Bhattacharyya et al., 2019 [15]	Dresden	-1.63 D (NS)	vs Control: increased (NS)	6 months	90% vs 0%
Ferdi et al. (Registry), 2023 [2]	Dresden	-3.7 D (p < 0.001)	NR	5 years	93%
Stojanovic et al., 2014 [58]	Dresden	-1.8 ± 1.4 D (p < 0.05)	vs A-CXL: +1.2 D (p < 0.05)	5 years	88% vs 74%

**Table 4:** Summary of keratometric outcomes (Kmax flattening, stabilization rate) from representative studies on transepithelial cross-linking protocols in progressive keratoconus D: diopter; Epi-off: epithelium-off protocols (conventional Dresden method); Epioxa™: FDA-approved epi-on CXL using oxygen-enriched RF for keratoconus; EpiSmart: investigational epi-on CXL with RiboStat (RF + sodium iodide); I-CXL: transepithelial corneal collagen crosslinking using iontophoresis (riboflavin delivery via iontophoresis); Kmax: maximum keratometry; NR: not reported; NS: not significant (p-value > 0.05); RF: riboflavin; TE-CXL: transepithelial corneal cross-linking; TPGS: tocopheryl polyethylene glycol succinate (water-soluble form of vitamin e)

Study	TE-CXL Protocol	Kmax Change	Comparison	Timepoint	Stabilization Rate
Al Fayez et al., 2015 [37]	Standard TE-CXL	+1.1 D (p < 0.05)	vs Dresden: -2.4 D (p < 0.0001)	3 years	41% vs 100%
Bikbova et al., 2016 [40]	I-CXL	-0.9 D (p < 0.05)	vs Dresden: -1.8 D (p < 0.05)	2 years	100% vs 100%
Caruso et al., 2016 [41]	Vitamin E-TPGS	-1.01 ± 1.22 D (p < 0.001)	NR	2 years	80%
Vinciguerra et al., 2016 [61]	I-CXL	-0.31 ± 1.87 D (NS)	vs Dresden: -1.05 D (p < 0.01)	1 year	NR
Stulting et al., 2018 [59]	Enhanced RF	-0.48 D (p < 0.001)	NR	2 years	100%
Epstein et al., 2022 [45]	EpiSmart	-0.76 D (p < 0.001)	NR	1 year	75%
Smith et al., 2025 [57]	Epioxa™ + O2	≤ -1.0 D (p < 0.05)	vs sham: increased (p < 0.0001)	1 year	NR

**Table 5:** Summary of keratometric outcomes (Kmax flattening, stabilization rate) from representative studies on accelerated cross-linking protocols in progressive keratoconus A-CXL: accelerated corneal cross-linking; ATE-CXL: accelerated transepithelial corneal cross-linking (30 mW/cm2 × 3 min); cl-ACXL: continuous light-accelerated corneal cross-linking (12 mW/cm2 × 10 min); D: diopter; Kmax: maximum keratometry (the steepest corneal curvature measurement in diopters); mW/cm2: milliwatts per square centimeter; NR: not reported; NS: not significant (p-value > 0.05); pl-ACXL: pulsed light-accelerated corneal cross-linking (30 mW/cm2 × 4 min); PRK: photorefractive keratectomy; TE-CXL: transepithelial corneal cross-linking

Study	A-CXL Protocol	Kmax Change	Comparison	Timepoint	Stabilization Rate
Iqbal et al., 2019 [77]	30 mW/cm2 × 8 min pulsed + PRK	-2.40 ± 0.69 D (p < 0.001)	vs Dresden: -1.62 D (p < 0.05)	1 year	100% vs 100%
Iqbal et al., 2019 [77]	30 mW/cm2 × 8 min pulsed + PRK	-2.23 ± 0.56 D (p < 0.001)	vs Dresden: -2.03 D (NS)	2 years	100% vs 100%
Marafon et al., 2020 [27]	30 mW/cm2 × 8 min pulsed	-0.90 ± 3.12 D (p < 0.001)	vs Dresden: -0.68 D (NS)	6 months	96% vs 90%
Badawi, 2021 [67]	10 mW/cm2 × 9 min	-1.87 ± 0.32 D (p < 0.0001)	vs Dresden: -2.74 D (NS)	1 year	NR
Badawi, 2021 [67]	10 mW/cm2 × 9 min	-1.87 ± 0.32 D (p < 0.0001)	vs TE-CXL: -0.36 D (p < 0.0001)	1 year	NR
Ishii et al., 2022 [78]	ATE-CXL	–0.48 D (NS)	NR	3 years	88%
Yousif et al., 2023 [94]	pl-ACXL	-0.61 ± 3.73 D (NS)	vs cl-ACXL: -0.36 D (NS)	1 year	NR
Kandel et al., 2024 [24]	9 mW/cm2 × 10 min	+1.2 ± 2.7 (NS)	vs Dresden: -1.8 D (p < 0.05)	5 years	73% vs 88%

Visual Acuity Outcomes

Overall, visual acuity was stabilized or modestly improved following corneal CXL across all protocols, with corrected distance visual acuity (CDVA) or best corrected visual acuity (BCVA) demonstrating more consistent and durable gains than uncorrected measures. Conventional epi-off CXL showed the most reliable long-term visual acuity stabilization, while accelerated and enhanced transepithelial approaches produced comparable short- to intermediate-term outcomes with faster postoperative recovery.

Visual acuity outcomes, including uncorrected distance visual acuity (UDVA) and CDVA/BCVA, were reported in 68 of the 84 included studies [2,7,12,14-18,20,22,24,25,27-30,32-42,44-50,52-54,57-61,63-75,77-81,83-85,87,91-94]. Across protocols, improvements were more consistently observed in CDVA than UDVA and most commonly emerged within the first six to 12 months following treatment, paralleling reductions in corneal irregularity and keratometric flattening [2,12,16,34,36,40,59].

Conventional epi-off CXL demonstrated the most durable visual acuity benefits, with sustained CDVA stabilization or improvement reported through two to five years and a low proportion of eyes experiencing visual decline in long-term registry and cohort data [24,34,36]. In contrast, transepithelial CXL exhibited more heterogeneous visual outcomes. Standard epi-on protocols were frequently associated with smaller or less durable CDVA gains beyond 12 months, whereas enhanced transepithelial approaches incorporating iontophoresis, modified riboflavin formulations, or supplemental oxygen achieved CDVA stabilization approaching epi-off outcomes in selected prospective studies [41,45,50,57,59].

Accelerated CXL protocols demonstrated visual acuity outcomes comparable to conventional epi-off CXL at short- and intermediate-term follow-up, with visual stability generally maintained through three to five years, although improvements often plateaued earlier in some cohorts [12,24,65,72-74].

Collectively, visual acuity outcomes across protocols mirrored keratometric trends, with conventional epi-off CXL providing the most durable long-term stabilization, while accelerated and enhanced transepithelial approaches demonstrated comparable short-term efficacy with advantages in recovery time. Quantitative visual acuity changes are reported in logMAR units as mean ± SD or median (IQR), as specified by the source studies. Reported p-values and 95% confidence intervals are derived from individual randomized trials, observational cohorts, or registry analyses where available, and outcomes stratified by follow-up interval are summarized in Table 6.

**Table 6:** Summary of visual acuity outcomes across corneal collagen cross-linking protocols in progressive keratoconus A-CXL: accelerated corneal cross-linking; BCVA: best corrected visual acuity; CDVA: corrected distance visual acuity; CXL: corneal collagen cross-linking; logMAR: logarithm of the minimum angle of resolution; NR: not reported; NS: not significant (p-value > 0.05); P: p-value; TE-CXL: transepithelial corneal cross-linking; UDVA: uncorrected distance visual acuity

Study	Protocol	UDVA Change	CDVA/BCVA Change	Timepoint
Wittig-Silva et al., 2014 [36]	Dresden	-0.15 ± 0.06 logMAR (p < 0.01)	-0.09 ± 0.03 logMAR ( p < 0.01)	3 years
Strmeňová et al., 2015 [34]	Dresden	NR	-0.048 logMAR (p < 0.01)	2 years
Bikbova et al., 2016 [40]	I-CXL	NS	-0.07 logMAR (p < 0.05)	2 years
Bikbova et al., 2016 [40]	Dresden	NS	-0.02 logMAR (p < 0.05)	2 years
Choi et al., 2017 [16]	Dresden	-0.09 ± 0.09 (p < 0.01)	NR	6 months
Choi et al., 2017 [16]	A-CXL	NS	NR	6 months
Stulting et al., 2018 [59]	TE-CXL	-0.146 logMAR (p < 0.0001)	-0.108 logMAR (p < 0.0001)	2 years
Kobashi et al., 2020 [12]	Dresden	NS	-0.02 logMAR (p < 0.0001)	1 year
Kobashi et al., 2020 [12]	A-CXL	NS	NS	1 year
Ferdi et al. (Registry), 2023 [2]	Dresden	-0.074 logMAR (p < 0.001)	NR	1 year
Ferdi et al. (Registry), 2023 [2]	Dresden	-0.138 logMAR (p < 0.001)	NR	5 years
Kandel et al. (Registry), 2024 [24]	Dresden	-0.204 logMAR (95% CI: -0.158 to -0.25)	-0.114 logMAR (95% CI: -0.07 to -0.156)	5 years
Kandel et al. (Registry), 2024 [24]	A-CXL	-0.098 logMAR (95% CI: -0.032 to -0.164)	-0.004 logMAR (95% CI: -0.044 to +0.05)	5 years

Endothelial Cell Density Outcomes

All corneal CXL protocols demonstrated a strong endothelial safety profile, with no clinically meaningful endothelial cell loss observed when established safety parameters were followed. Across studies, mean ECD changes remained within physiologic measurement variability over short- and long-term follow-up.

ECD was reported in 52 of the 84 included studies and consistently demonstrated preservation across conventional, accelerated, and transepithelial CXL protocols, with no cases of endothelial decompensation reported during follow-up [2,7,12,14-18,20,22,24,25,27,28,30,32-42,44,45,47,49,50,52,57-61,63,65,66,69,72-75,78,80,83-85,91,93]. Across RCTs, comparative cohorts, and large registries, mean ECD changes were minimal and not clinically significant, with values remaining within expected test-retest variability [2,14-16,34,36,40].

Conventional epi-off (Dresden) CXL showed stable endothelial outcomes across short-, intermediate-, and long-term follow-up, with no consistent or statistically significant deviations from baseline reported in randomized trials, observational cohorts, or registry-based analyses [2,14-16,24,34,36,40,58]. Transepithelial (epi-on) CXL protocols demonstrated similarly favorable endothelial safety, with prospective and comparative studies reporting equivalent endothelial preservation and no clinically meaningful differences compared with epi-off approaches [7,14,37,40,42,45,50,58,61]. Enhanced transepithelial techniques, including oxygen supplementation and iontophoresis-assisted riboflavin delivery, maintained endothelial stability through intermediate follow-up [41,45,50,60].

Accelerated CXL protocols exhibited endothelial safety comparable to conventional Dresden CXL. Comparative trials and meta-analyses reported no significant differences in ECD change between accelerated and standard protocols at short-term follow-up, with longer-term cohort and registry data confirming sustained endothelial preservation through three to five years, including with pulsed and high-fluence variants [12,24,72-74,78,81,84]. Rare reports of notable ECD reduction were confined to thin corneas or hypo-osmolar riboflavin use and did not result in endothelial dysfunction [83].

Across all protocols, no consistent associations were identified between baseline age, keratoconus severity, or treatment intensity and clinically meaningful endothelial cell loss [2,59,61]. Collectively, these findings support a robust endothelial safety profile for conventional, accelerated, and transepithelial CXL when performed within established safety thresholds. Quantitative ECD outcomes are summarized in Table 7 as mean ± SD (cells/mm²), with reported p-values and confidence intervals reflecting within-study comparisons and registry-based analyses rather than pooled estimates.

**Table 7:** Summary of endothelial cell density (ECD) outcomes from representative studies across protocols in progressive keratoconus A-CXL: accelerated corneal cross-linking; CXL: corneal collagen cross-linking; ECD: endothelial cell density (the number of endothelial cells per square millimeter in the cornea); TE-CXL: transepithelial corneal cross-linking

CXL Protocol	Mean Baseline ECD (Range)	Mean Follow-up ECD (Range)	Mean Change (Range)	Mean Follow-Up (Range)	Statistical Significance
Dresden [14-16,34-37,40,50,58,72,73,75,85,91]	2697.8 cells/mm2 (2690–2846)	2682 cells/mm2 (2672–2805)	-15.8 cells/mm2 (-33 to +3)	17.5 months (6–40)	No significant differences in ECD were observed within or between protocols (p > 0.05).
TE-CXL [14,37,40,42,45,50,51,58,61]	2663 cells/mm2 (2545–2750)	2651.6 cells/mm2 (2532–2738)	-13.2 cells/mm2 (-100 to -12)	21.1 months (12–40)	No significant differences in ECD were observed within or between protocols (p > 0.05).
A-CXL [16,62,72,73,75,80,85,91,93]	2772.3 cells/mm2 (2485–2854)	2742.9 cells/mm2 (2468–2821)	-29.4 cells/mm2 (-15 to -12)	11.5 months (6–24)	No significant differences in ECD were observed within or between protocols (p > 0.05).

Safety and Complications

Across all protocols, corneal CXL demonstrated a favorable safety profile, with adverse events predominantly transient and serious vision-threatening complications remaining rare. Differences in safety outcomes were largely driven by epithelial management and postoperative stromal remodeling rather than treatment fluence alone.

Transient and self-limited adverse events: Transient postoperative adverse events were common and protocol-dependent. Conventional epi-off CXL was associated with the highest incidence of early corneal haze, particularly within the first one to three months, with reported rates ranging widely across studies but declining substantially over time. Large registry data demonstrated haze rates of approximately 15% at one year and <2-3% by five years, with persistent visually significant haze remaining uncommon [2,24]. Postoperative pain, delayed epithelial healing, and sterile infiltrates were more frequent following epi-off protocols but were typically self-limited and responsive to standard management [7,16,19,32].

Transepithelial and accelerated CXL protocols demonstrated more favorable short-term tolerability, with lower rates of corneal haze, fewer epithelial healing complications, reduced postoperative discomfort, and faster early visual recovery compared with conventional epi-off CXL [7,14,37,44,47]. Comparative cohorts and meta-analyses reported similar or reduced transient adverse event rates with accelerated protocols relative to Dresden CXL, without an increase in long-term complications [12,72-74,81].

Serious and vision-threatening complications: Serious vision-threatening complications following CXL were rare across all protocols. Infectious keratitis occurred infrequently, predominantly after epi-off treatment, with large retrospective series reporting incidence rates on the order of 0.001-0.5% [56]. Central toxic keratopathy was reported primarily following accelerated protocols in isolated series and remained uncommon overall [70]. Other serious events, including persistent epithelial defects, stromal scarring, or retreatment for progression, were rare and occurred more frequently in high-risk corneas or modified protocols [21,24,78].

Overall, when performed within established safety parameters, all CXL protocols demonstrated acceptable safety profiles. Epi-off CXL was associated with higher rates of transient haze and discomfort but low long-term morbidity, while transepithelial and accelerated approaches offered improved early tolerability with similarly low rates of serious complications (Tables 8, 9).

**Table 8:** Summary of corneal haze rates across corneal collagen cross-linking protocols in progressive keratoconus A-CXL: accelerated corneal cross-linking; CXL: corneal collagen cross-linking; I-CXL: transepithelial corneal collagen crosslinking using iontophoresis (riboflavin delivery via iontophoresis); NR: not reported

Study	CXL Protocol	Haze Rate	Severity	Resolution
Kortuem et al., 2017 [81]	Dresden	70.5%	Variable	Progressive improvement
Kortuem et al., 2017 [81]	A-CXL	46.9%	Variable	Progressive improvement
Bhattacharyya et al., 2019 [15]	Dresden	All patients	Transient	Resolved by 3 months
Ferdi et al. (Registry), 2023 [2]	Dresden	15.1% at 1 year	Variable	NR
Ferdi et al. (Registry), 2023 [2]	Dresden	1.9% at 5 years	Variable	NR
Kandel et al. (Registry), 2024 [24]	Dresden	3% at 5 years	Variable	Resolution in 77%
Kandel et al. (Registry), 2024 [24]	A-CXL	2% at 5 years	Variable	Resolution in 88%
Alqudah et al., 2025 [14]	I-CXL	7%	Mild	NR
Alqudah et al., 2025 [14]	Dresden	54% mild	Mild	NR

**Table 9:** Summary of other complications from representative studies across corneal collagen cross-linking protocols in progressive keratoconus A-CXL: accelerated corneal cross-linking; ATE-CXL: accelerated transepithelial corneal cross-linking (a high-intensity, short-duration epi-on protocol); CXL: corneal collagen cross-linking; ECD: endothelial cell density (the number of endothelial cells per square millimeter in the cornea); RF: riboflavin; TE-CXL: transepithelial corneal cross-linking

Complication	Study	CXL Protocol	Rate	Notes
Corneal scarring	Ferdi et al., 2019 [21]	Dresden	3.0% at 1 year	Declining over time
Endothelial cell loss	Manumuraleekrishna et al., 2024 [83]	A-CXL (Hypo-osmolar RF)	1 eye	Significant ECD loss
Microbial keratitis	Kandel et al. (Registry), 2024 [24]	A-CXL	1 case	Led to scarring
Need for re-treatment	Ishii et al., 2022 [78]	ATE-CXL	2 eyes	Due to progression
Persistent epithelial defect	Ferdi et al., 2019 [21]	TE-CXL	1.40%	Overall rate
Sterile infiltrates	Kandel et al. (Registry), 2024 [24]	A-CXL	Reported	Resolved with treatment
Stromal edema	Bikbova et al., 2016 [40]	Dresden	Observed	Resolved 6-12 months

Corneal Thickness Changes (Pachymetry)

All corneal CXL protocols exhibited a predictable biphasic pachymetric response, characterized by early postoperative thinning followed by partial or complete recovery within the first year. No protocol was associated with progressive long-term corneal thinning when established safety thresholds were respected.

Corneal thickness changes, assessed primarily by central corneal thickness (CCT) or thinnest corneal thickness (TCT), were reported in 48 of the 84 included studies [2,7,12,14,15,17,18,20-22,24,25,27,28,30,32,33,36,37,39-44,46-51,55,58,60,61,63,65,69,71-74,76,80,81,91,92,94]. Across protocols, studies consistently demonstrated transient early thinning attributable to stromal dehydration and compaction, followed by recovery toward baseline within 3-12 months, without association with endothelial compromise or ectatic progression [2,12,14,15,36,61].

Conventional epi-off (Dresden) CXL was associated with greater early pachymetric reduction compared with transepithelial approaches, with recovery observed over subsequent months and long-term stability thereafter [2,14,15,20,21,36]. Large registry and long-term cohort data confirmed minimal net pachymetric change at 3-5 years, supporting the absence of persistent stromal thinning following standard CXL [2,24]. Use of hypo-osmolar riboflavin in thin corneas preserved safety while mitigating excessive early thinning [72,76].

Transepithelial (epi-on) CXL demonstrated smaller and shorter-lived pachymetric changes, with rapid recovery reflecting limited stromal dehydration and preserved epithelial integrity [7,37,39,41,43,44,51,61]. Comparative studies reported significantly less early thinning with epi-on techniques than with epi-off CXL, without meaningful long-term differences in corneal thickness [7,43,44,61]. Enhanced transepithelial protocols maintained pachymetric stability through intermediate follow-up [46-50,60].

Accelerated CXL protocols exhibited pachymetric responses comparable to conventional epi-off CXL, characterized by early thinning followed by recovery within several months [55,63,65,91]. Comparative trials and meta-analyses demonstrated no significant long-term differences in CCT or TCT between accelerated and standard protocols, including pulsed and customized variants [12,17,25,69,71-74,80,81,94].

Across all protocols, early pachymetric reduction correlated with stromal remodeling and transient haze formation but did not predict long-term corneal instability or visual decline [2,59,61]. Overall, corneal thickness changes following CXL were transient and protocol dependent, with recovery toward baseline observed in the majority of eyes and no evidence of progressive thinning when established safety parameters were followed. Quantitative corneal thickness outcomes are summarized in Table 10 as mean ± SD, with reported p-values reflecting individual study-level analyses rather than pooled estimates.

**Table 10:** Summary of pachymetry outcomes (corneal thickness changes) from representative studies across cross-linking protocols in progressive keratoconus A-CXL: accelerated corneal cross-linking; CCT: central corneal thickness; CXL: corneal collagen cross-linking; I-CXL: transepithelial corneal collagen crosslinking using iontophoresis (riboflavin delivery via iontophoresis); NR: not reported; NS: not significant (p-value > 0.05); TCT: thinnest corneal thickness

Study	CXL Protocol	Baseline CCT/TCT	Change	Timepoint	Significance
Wittig-Silva et al., 2014 [35]	Dresden	NR	-19.52 ± 5.06 μm	3 years	p < 0.001
Wittig-Silva et al., 2014 [35]	Control	NR	-17.01 ± 3.63 μm	3 years	p < 0.001
Vinciguerra et al., 2016 [60]	Dresden	NR	-41.1 ± 35.3 μm	1 year	p < 0.001
Vinciguerra et al., 2016 [60]	I-CXL	NR	+1.0 ± 7.2 μm	1 year	NS
Kortuem et al., 2017 [81]	Dresden	457.75 μm	Progressive thinning	3 years	p < 0.05
Kandel et al. (Registry), 2024 [24]	Dresden	NR	-3.0 μm	5 years	NS
Kandel et al. (Registry), 2024 [24]	A-CXL	NR	-11.8 μm	5 years	p < 0.001
Ferdi et al. (Registry), 2023 [2]	Dresden	459 μm	-17 μm	1 year	p < 0.001
Ferdi et al. (Registry), 2023 [2]	Dresden	459 μm	-11 μm	5 years	p < 0.05

Biomechanical and Surrogate Outcomes

Surrogate biomechanical measures supported effective stromal cross-linking across protocols, with greater treatment penetration generally observed following epi-off techniques. However, these metrics demonstrated variable correlation with long-term clinical outcomes and should be interpreted as complementary rather than definitive efficacy endpoints.

Biomechanical and surrogate outcomes, including stromal demarcation line depth, corneal hysteresis (CH), corneal resistance factor (CRF), and deformation-based parameters derived from devices such as the Ocular Response Analyzer® (Reichert, Inc., Depew, New York, United States) and Corvis® ST (OCULUS Optikgeräte GmbH, Wetzlar, Germany), were reported in 32 of the 84 included studies [7,12,14,16-18,21-25,39-42,50,53-55,58,61,64,67,69,74,75,80,83,88,90,94]. These measures were primarily used as indirect indicators of stromal cross-linking effect and biomechanical stiffening rather than direct clinical endpoints.

Conventional epi-off (Dresden) CXL consistently demonstrated deeper stromal demarcation lines and more pronounced biomechanical changes compared with transepithelial approaches, reflecting greater riboflavin penetration and treatment depth [21,25,39]. While modest increases in CH and CRF were reported in several studies, findings were variable and not uniformly significant, whereas deformation-based parameters more consistently indicated increased biomechanical stability following treatment [21,74].

Transepithelial (epi-on) CXL generally produced shallower demarcation lines and less consistent biomechanical changes, consistent with reduced riboflavin diffusion through the intact epithelium [39,61]. Enhanced transepithelial techniques incorporating oxygen supplementation, iontophoresis, or modified riboflavin formulations achieved greater stromal penetration in selected studies, with biomechanical profiles approaching those of epi-off protocols [41,52]. Nonetheless, biomechanical changes following standard epi-on CXL remained heterogeneous, paralleling the variability observed in long-term clinical outcomes [7,40].

Accelerated CXL protocols exhibited biomechanical surrogate outcomes broadly comparable to conventional epi-off CXL when equivalent total fluence was delivered [12,17,69,80]. Comparative trials and meta-analyses reported no consistent differences in demarcation depth or biomechanical parameters between accelerated and standard protocols, although higher irradiance regimens were occasionally associated with slightly reduced penetration [12]. Pulsed and adapted-fluence accelerated variants demonstrated similar biomechanical profiles, potentially reflecting improved oxygen availability during treatment [80,94].

Across all protocols, greater demarcation line depth correlated with baseline corneal thickness and disease severity but did not reliably predict long-term keratometric or visual outcomes [12,25,61]. Cross-study comparability was limited by variability in measurement techniques, device-specific parameters, and follow-up duration. Overall, biomechanical and surrogate measures provided supportive evidence of effective stromal cross-linking but should be interpreted as adjunctive indicators alongside clinical endpoints. Quantitative biomechanical outcomes are summarized in Tables 11, 12 using the statistical format reported by the source studies, with p-values and confidence intervals reflecting individual study-level or meta-analytic analyses where applicable.

**Table 11:** Summary of corneal stromal demarcation line depth outcomes from representative studies across cross-linking protocols in progressive keratoconus A-CXL: accelerated corneal cross-linking; AS-OCT: anterior segment optical coherence tomography; cl-ACXL: continuous light-accelerated corneal cross-linking; CXL: corneal collagen cross-linking; EFPL-M-TECXL: transepithelial enhanced fluence pulsed light accelerated CXL; eRF: enhanced riboflavin; I-CXL: transepithelial corneal collagen crosslinking using iontophoresis (riboflavin delivery via iontophoresis); μm: micrometer; NR: not reported; NS: not significant (p-value > 0.05); pl-ACXL: pulsed light-accelerated corneal cross-linking; RF: riboflavin; WMD: weighted mean difference

Study	CXL Protocol	Demarcation Line Depth (via AS-OCT)	Comparison
Bikbova et al., 2016 [39]	I-CXL	172 ± 16 μm	vs Dresden: 292 μm (p < 0.001)
Dervenis et al., 2020 [17]	Dresden	322.50 μm	vs pl-ACXL: 319.95 μm (NS)
Kobashi et al., 2020 [12]	Dresden	Deeper than A-CXL (actual values NR)	WMD vs A-CXL: -102.25 μm 95% CI: -157.16 to -47.35 (p < 0.001)
Mazzotta et al., 2022 [52]	TE-EFPL ACXL	282.6 ± 23.6 μm	NR
Karotkar et al., 2022 [80]	pl-ACXL	251.13 ± 18.28 μm	vs cl-ACXL: 245.28 μm (NS)
Manumuraleekrishna et al., 2024 [83]	A-CXL (Iso-osmolar RF)	252 μm	vs hypo-osmolar: 210 μm (NS)
Dina et al., 2025 [69]	A-CXL	278.9 ± 31.71 μm	vs Dresden: 280.42 μm (NS)

**Table 12:** Summary of other biomechanical parameter outcomes from representative studies across cross-linking protocols in progressive keratoconus A-CXL: accelerated corneal cross-linking; CH: corneal hysteresis (viscoelastic property of the cornea; surrogate for corneal biomechanical strength); cl-ACXL: continuous light-accelerated corneal cross-linking; CRF: corneal resistance factor (a measure of viscous and elastic resistance of the cornea); CXL: corneal collagen cross-linking; DAI: deformation amplitude index (normalized corneal stiffness relative to intraocular pressure); DAR: deformation amplitude ratio (corneal stiffness); IR: integrated radius (corneal resistance to deformation); MHz: megahertz; NS: not significant (p-value > 0.05); pl-ACXL: pulsed light-accelerated corneal cross-linking; RF: riboflavin; TE-CXL: transepithelial corneal cross-linking

Study	CXL Protocol	Parameter	Timepoint	Change	Significance
Hashemi et al., 2015 [74]	A-CXL vs Dresden	CH	6 months	No significant difference	NS
Hashemi et al., 2015 [74]	A-CXL vs Dresden	CRF	6 months	No significant difference	NS
Shao et al., 2020 [55]	TE-CXL	Brillouin shift	6 months	+25 MHz cone region	p < 0.01
Karotkar et al., 2022 [80]	cl-ACXL	DAI	1 year	-0.19 mm (1.04→0.85)	p < 0.05
Karotkar et al., 2022 [80]	pl-ACXL	DAI	1 year	-0.28 mm (1.12→0.84)	p < 0.01
Manumuraleekrishna et al., 2024 [83]	A-CXL (Iso- vs Hypo-osmolar RF)	CH	1 year	Greater in iso-osmolar RF	p < 0.05
Manumuraleekrishna et al., 2024 [83]	A-CXL (Iso- vs Hypo-osmolar RF)	CRF	1 year	Greater in iso-osmolar RF	p < 0.05
Ang et al., 2025 [64]	A-CXL	DAR	6 months	-1.447	p < 0.01
Ang et al., 2025 [64]	A-CXL	IR	6 months	-1.753	p < 0.05

Discussion

Summary of Key Findings

This systematic review synthesizes comparative evidence from 84 studies evaluating conventional Dresden (epi-off), accelerated, and transepithelial (epi-on) corneal CXL protocols for progressive keratoconus. Across randomized trials, observational cohorts, and large registry datasets, conventional epi-off CXL consistently demonstrated the most durable keratometric stabilization and visual acuity preservation, with sustained effects reported up to 5-10 years in long-term follow-up [2,20,24,36]. Accelerated protocols achieved comparable short-term outcomes but exhibited greater variability in durability beyond two to three years, particularly in registry-based analyses [12,24]. In contrast, transepithelial approaches showed more heterogeneous efficacy, with standard epi-on protocols frequently underperforming relative to epi-off CXL unless enhanced with modified riboflavin delivery or supplemental oxygen [37,40,45,57].

Despite these differences in efficacy, safety profiles across all protocols were favorable. ECD was preserved, corneal thickness changes followed predictable and transient biphasic patterns, and serious complications remained rare [2,7,12,24]. Collectively, these findings suggest that differences in long-term outcomes are driven primarily by epithelial management, oxygen availability, and effective stromal penetration depth rather than by treatment fluence alone, underscoring the importance of protocol selection tailored to disease severity and patient-specific factors.

Reconciling Differences in Protocol Efficacy

Observed heterogeneity in CXL efficacy across protocols reflects fundamental differences in epithelial removal, oxygen-dependent photochemical reactions, and stromal penetration rather than total delivered fluence alone [3,5,12]. Conventional epi-off CXL enables unimpeded riboflavin diffusion and sustained oxygen availability, resulting in deeper and more uniform stromal cross-linking. These mechanistic advantages are consistent with its superior long-term keratometric stability and visual outcomes [17,39,69].

Accelerated CXL protocols, while grounded in the Bunsen-Roscoe reciprocity law, may be constrained by oxygen depletion at higher irradiances. This limitation likely contributes to the greater variability in long-term outcomes observed in registry and cohort data, despite short-term non-inferiority demonstrated in randomized trials and meta-analyses [3,12,24]. Pulsed and adapted-fluence accelerated strategies partially address this limitation by facilitating oxygen replenishment during treatment and have demonstrated biomechanical and clinical outcomes approaching those of conventional protocols in selected studies [69,80,94].

Historically, transepithelial CXL underperformed due to the epithelial barrier limiting riboflavin diffusion, resulting in shallower demarcation depths and reduced biomechanical stiffening [39,61]. However, recent enhancements-including oxygen supplementation, iontophoresis, and modified riboflavin formulations-have improved stromal penetration and surrogate biomechanical measures, narrowing the efficacy gap with epi-off CXL in prospective trials [41,45,52,57]. These advances highlight the evolving nature of epi-on techniques and the importance of distinguishing standard from enhanced transepithelial protocols in comparative analyses.

Safety Considerations Across CXL Techniques

Across all protocols, CXL demonstrated a favorable safety profile, with most adverse events being transient and closely related to epithelial management and postoperative stromal remodeling [2,7,12]. Conventional epi-off CXL was associated with higher rates of early corneal haze and postoperative discomfort; however, haze typically resolved over time and rarely progressed to visually significant scarring, resulting in low long-term morbidity [2,15,21,24]. Accelerated and transepithelial protocols generally offered improved early tolerability, with lower rates of epithelial complications and faster visual recovery [7,37,50,72].

Serious complications-including infectious keratitis, central toxic keratopathy, and persistent stromal scarring-were rare across all protocols and were most commonly reported in the context of epi-off or high-risk accelerated treatments accompanied by additional risk factors [24,56,70]. These findings reinforce the overall safety of CXL when established parameters are followed and emphasize the importance of careful patient selection, perioperative management, and adherence to protocol-specific safety thresholds.

Clinical Implications and Protocol Selection

From a clinical standpoint, these findings support conventional epi-off CXL as the preferred option for patients with moderate-to-advanced keratoconus or a high risk of progression, where durable long-term stabilization is paramount [2,24,36]. Accelerated protocols represent a reasonable alternative when treatment efficiency, patient tolerance, or resource constraints are prioritized, particularly given their comparable short-term efficacy [12,24,72]. Enhanced transepithelial approaches may be appropriate for select patients seeking reduced invasiveness and postoperative discomfort, provided that potential trade-offs in long-term durability are carefully considered [37,45,57].

Accordingly, shared decision-making that integrates baseline disease severity, corneal thickness, progression risk, and patient preferences remains essential, especially as protocol refinements and longer-term comparative data continue to emerge.

Limitations

This review is subject to several limitations. Substantial heterogeneity in study design, outcome definitions, follow-up duration, and protocol parameters precluded quantitative meta-analysis. Long-term comparative data beyond five years remain limited for accelerated and transepithelial protocols, and many studies relied on surrogate biomechanical measures rather than direct clinical endpoints [12,21]. These limitations should be considered when interpreting comparative efficacy, particularly for newer or evolving protocol variants.

## Conclusions

This systematic review of 84 studies published through December 2025 supports conventional Dresden epi-off corneal CXL as the most consistently supported reference standard for the management of progressive keratoconus in adults, with robust evidence for long-term keratometric stabilization, visual acuity preservation, biomechanical reinforcement, and endothelial safety. Accelerated CXL protocols demonstrate comparable short-term efficacy with advantages in treatment efficiency, reduced postoperative morbidity, and faster visual recovery, supporting their use as appropriate alternatives in selected clinical settings.

Enhanced transepithelial (epi-on) approaches, including oxygen-augmented and modified riboflavin delivery systems, offer improved tolerability and safety with minimal invasiveness and may be suitable for early-stage disease, thin corneas, or patients prioritizing comfort. However, long-term durability remains more variable without protocol optimization. Collectively, these findings emphasize the importance of individualized, patient-tailored protocol selection and standardized monitoring strategies to optimize outcomes and address the growing global burden of keratoconus. Further well-designed RCTs with extended follow-up and broader population representation are needed to refine protocol selection and clarify long-term comparative durability.

## Appendices

Appendix A: large language model-assisted search process

To supplement the manual systematic search and address potential limitations in identifying comprehensive data on CXL protocols, a large language model (LLM) was employed to screen over 200 million academic papers from the Semantic Scholar corpus. This automated process applied predefined inclusion criteria to identify additional relevant sources: Progressive keratoconus population (e.g., documented progression via ≥1 D Kmax increase over 6-12 months); Comparative design evaluating at least two CXL protocols (conventional Dresden, accelerated, or transepithelial/epi-on); Well-defined standalone CXL interventions (excluding combinations like intracorneal rings); Minimum 12-month follow-up; Relevant outcomes (e.g., keratometric stabilization, visual acuity, endothelial cell density, complications); Adult patients (≥18 years) with ≥10 per group; Published between January 1, 2013, and December 26, 2025.

The LLM assigned relevance ranking scores, yielding 376 additional sources (including 49 duplicates), which were integrated and deduplicated for screening.

Appendix B: detailed instructions for LLM-assisted data extraction

Data extraction used an LLM to retrieve structured information from the 84 included studies, with manual verification for accuracy. The LLM was prompted with column-specific instructions, focusing on full-text content where available, and providing quotes, table/figure references, and explanations. Outputs were compiled into tables; incomplete data were marked "Not reported." The extracted columns and instructions were:

*Study Characteristics*: Extract study design (e.g., RCT, cohort), setting (country, institution), period, sample size per group, randomization/blinding, and primary objective.

*Population Definition*: Extract progression criteria (e.g., Kmax increase), baseline severity, age (mean/range), inclusion/exclusion criteria, baseline corneal thickness, and comorbidities/treatments.

*CXL Protocol Details*: Extract protocol type, riboflavin composition/concentration, epithelial removal (if applicable), UV-A irradiance (mW/cm²), exposure time/energy dose (J/cm²), light delivery (pulsed/continuous), modifications, pre-/post-treatment procedures.

*Visual and Keratometric Outcomes*: Extract UDVA/BCVA baseline/changes (logMAR), keratometric indices (Kmax, Kmean), measurement methods, results with significance, stabilization definition, and follow-up timepoints.

*Safety and Endothelial Results*: Extract endothelial cell density baseline/changes, measurement method, complication rates/types (e.g., haze, edema, infection), resolution time, and pain scores.

*Follow-up Data*: Extract duration/timepoints, loss to follow-up (rates/reasons), completion rates, deviations/crossovers, statistical methods for missing data, analysis type (ITT/per-protocol).

*Change in Corneal Thickness (CCT/Thinnest Pachymetry)*: Extract mean ± SD/median [IQR] changes (µm) at timepoints, group differences (p-values), measurement method; prioritize thinnest point/CCT.

*Biomechanical/Surrogate Outcomes (Demarcation Line, CH, etc.)*: Extract demarcation line depth (µm, method), CH/CRF, other parameters (e.g., deformation amplitude), baseline/changes, group differences.

*Risk of Bias Assessment*: Use RoB 2 (RCTs) or ROBINS-I (non-randomized); summarize overall risk and key domains; based on reported information only, with quotes/references.
